# Artificial Intelligence-Based Classification of CT Images Using a Hybrid SpinalZFNet

**DOI:** 10.1007/s12539-024-00649-4

**Published:** 2024-08-21

**Authors:** Faiqa Maqsood, Wang Zhenfei, Muhammad Mumtaz Ali, Baozhi Qiu, Naveed Ur Rehman, Fahad Sabah, Tahir Mahmood, Irfanud Din, Raheem Sarwar

**Affiliations:** 1 School of Computer and Artificial Intelligence, Zhengzhou University, Zhengzhou, 450001 China; 2 Beijing University of Technology, Beijing, 100124 China; 3 Division of Electronics and Electrical Engineering, Dongguk University, Seoul, 04620 South Korea; 4 Department of Computer Science, New Uzbekistan University, Tashkent, 100174 Uzbekistan; 5 OTEHM, Faculty of Business and Law, Manchester Metropolitan University, M15 6BH Manchester, UK

**Keywords:** SpinalNet, Zeiler and Fergus network, Efficient neural network, Median filter, Computed tomography

## Abstract

The kidney is an abdominal organ in the human body that supports filtering excess water and waste from the blood. Kidney diseases generally occur due to changes in certain supplements, medical conditions, obesity, and diet, which causes kidney function and ultimately leads to complications such as chronic kidney disease, kidney failure, and other renal disorders. Combining patient metadata with computed tomography (CT) images is essential to accurately and timely diagnosing such complications. Deep Neural Networks (DNNs) have transformed medical fields by providing high accuracy in complex tasks. However, the high computational cost of these models is a significant challenge, particularly in real-time applications. This paper proposed SpinalZFNet, a hybrid deep learning approach that integrates the architectural strengths of Spinal Network (SpinalNet) with the feature extraction capabilities of Zeiler and Fergus Network (ZFNet) to classify kidney disease accurately using CT images. This unique combination enhanced feature analysis, significantly improving classification accuracy while reducing the computational overhead. At first, the acquired CT images are pre-processed using a median filter, and the pre-processed image is segmented using Efficient Neural Network (ENet). Later, the images are augmented, and different features are extracted from the augmented CT images. The extracted features finally classify the kidney disease into normal, tumor, cyst, and stone using the proposed SpinalZFNet model. The SpinalZFNet outperformed other models, with 99.9% sensitivity, 99.5% specificity, precision 99.6%, 99.8% accuracy, and 99.7% F1-Score in classifying kidney disease.

**Graphical Abstract:**

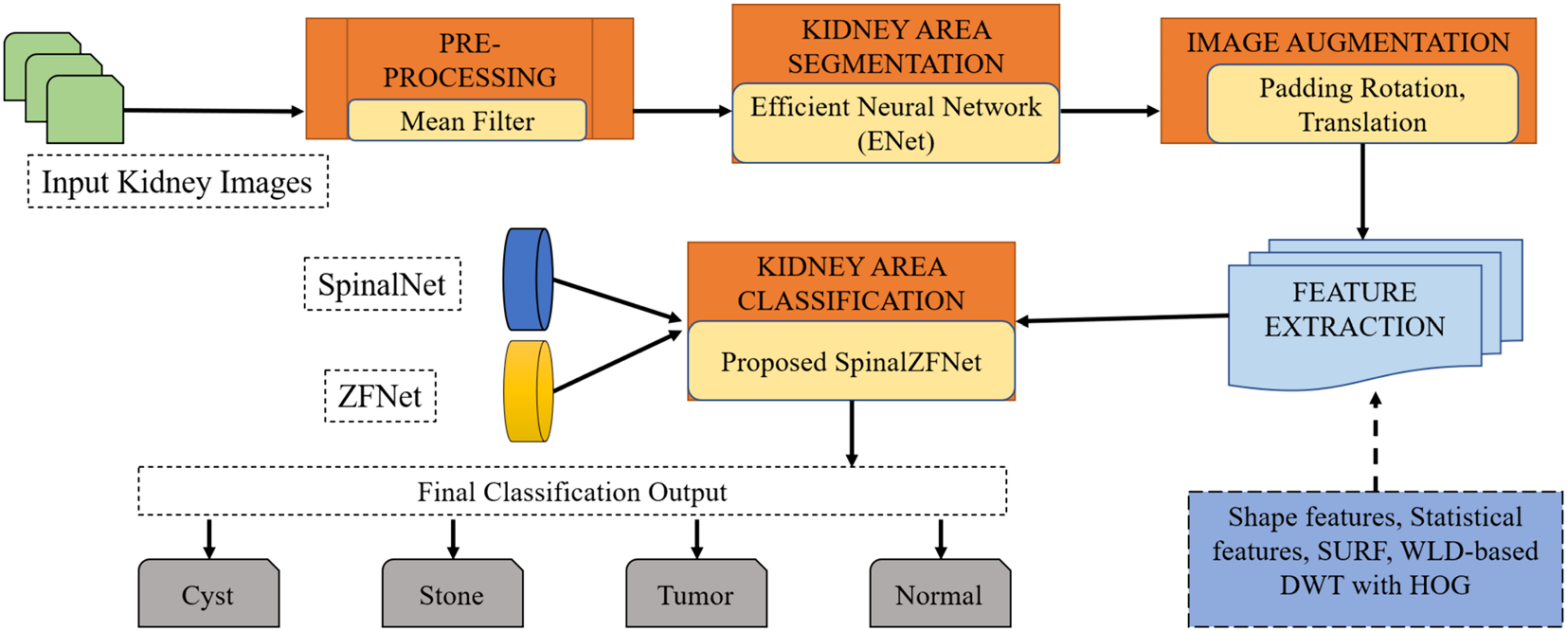

## Introduction

Worldwide, there has been an apparent increase in acute syndromes and infections, necessitating suitable clinical procedures to detect and treat these diseases immediately. If these diseases are left untreated, they will lead to serious health issues and death, resulting in a significant load on the healthcare system. The severity and mortality rate of acute diseases are much higher than that of infectious diseases [1]. The kidney is an abdominal organ that carries out the process of filtering the excess water and waste from the blood. Globally, over one-tenth of the population [2] is affected by the abnormalities that are developed in the kidneys, which also have the potential to cause an adverse effect on health. The precise cause of these abnormalities has not been completely recognized. However, several factors, such as lifestyle, environment, and genetics, have been determined to contribute to kidney abnormality in recent years [3]. These abnormalities are found to be commonly occurring in all individuals, irrespective of gender or age. Many abnormalities, including hydronephrosis, cysts, and stones, can affect the kidneys. While these conditions can be cured, if left untreated, they may lead to chronic kidney diseases such as cardiorenal syndrome, uremia, or kidney tumors [4]. Kidney cancer is one of the most common types of cancer worldwide, characterized by the abnormal growth of cells in the kidney. Kidney stones, which affect 12% of the population, are formed by the concretions of crystals. Cysts, on the other hand, are fluid-filled pockets surrounded by thin walls on the kidney surface [5].

As kidney abnormalities become more severe, symptoms such as swollen feet and legs, blood in urine, and pain in the back or sides may occur. Abnormalities, such as kidney tumor (renal cell carcinoma), kidney stones (nephrolithiasis), and cysts, can impair the normal functioning of the kidney [3]. The presence of tumor abnormalities can progress into severe conditions, such as chronic kidney diseases (CKD), a side effect of repeated stone abnormalities. The individuals with a high risk of stones are associated with an imminent risk of CKD and thus require dialysis. Hence, it is essential to identify kidney diseases at the primary stages to avoid serious complications [6]. Accordingly, the present diagnostic methods and patient management can be improved using Artificial Intelligence (AI) systems, which perform localization, classification, and quantification of kidney abnormalities based on images. Various imaging techniques, like positron emission tomography (PET), magnetic resonance imaging (MRI), computed tomography (CT), ultrasonography, X-rays, and other urography methods are used for capturing kidney images. Among these, CT scans are widely employed in detecting kidney diseases. However, every modality has strengths and weaknesses, resulting in distinct technical challenges while applying them [7]. Early detection is significant in avoiding severe complications at a later period. However, few radiologists and nephrologists are available worldwide, making timely detection challenging [5]. Further, accurate disease detection depends on the nephrologists’ experience in the manual examination. This process is highly subjective, error-prone, and time-consuming, thus requiring the use of the computer-aided diagnosis (CAD) technique for detection [8, 9].

Identifying kidney diseases is a multidisciplinary domain that experts in information technology investigate to assist medical professionals by creating a model of the biological process occurring in kidneys and generating consistent diagnostic outcomes. In recent years, an increased occurrence of imaging datasets has been noted due to the ability to store massive data and the coincidence of sophisticated machine learning (ML) and deep learning (DL) methods [10]. Recently, ML techniques have been employed to analyze diseases efficiently by discovering the people at risk in the early stages. ML-based algorithms are currently the most extensively used approach to kidney disease detection [11]. These techniques learn and improve when vast amounts of data are integrated to learn the decision rules explicitly. DL approaches have also contributed to the sophisticated progress in AI, and these methods involve tuning multi-layer artificial neural networks (ANN) on massive databases [12, 13]. The incorporation of DL and computer vision schemes has been successfully applied in various medical domains, such as lesion detection [14] classification [15], and imaging segmentation. Medical professionals use robust approaches to detect cancer and cardiac diseases [16]. Recently, convolutional neural networks (CNNs), a DL approach, have enhanced the accuracy of various computer vision processes, like semantic segmentation, object detection, and image recognition. The CNNs can also capture complex tissue patterns successively and have been extensively applied in biomedical imaging for segmentation and classification processes [17].

The contribution of this research is as follows:

i) A hybrid SpinalZFNet technique is proposed to classify kidney disease accurately from CT images. The SpinalZFNet was devised as a combination of SpinalNet and Zeiler Fergus Network (ZFNet).
ii) Preprocess the images using a median filter to remove the noise from the data. The preprocessed image is segmented using an Efficient Neural Network (ENet).
iii) For feature extraction, different methods are used, such as weber local descriptor (WLD) based discrete wavelet transform (DWT) with histogram of oriented gradients (HOG) extracted used for feature extractors. The kidney disease is finally classified from the extracted features into normal, cyst-tumor, and stone using the designed SpinalZFNet model.
iv) The performance of SpinalZFNet is evaluated based on the following: Sensitivity, Specificity, Precision, Accuracy, F1-Score, and Confusion matrix.
v) A comparative analysis was conducted comparing VGG + DN with KNN, DenseAUXNet201, MLP-ANN, and DRDC. The proposed model achieved a better 99.8% accuracy, 99.9% sensitivity, 99.5% specificity, 99.6% precision, and 99.7 F1-Score than the state-of-the-art models.

The paper workflow is as follows: Sect. 2 describes the related work overview. Section 3 presents detailed explanations of our proposed method. Section 4 describes the experimental datasets, evaluation metrics, and results. Section 5 discusses our findings. The conclusion is provided in Sect. 6.

## Literature Review

This section summarizes the literature on different prevailing methods used for kidney cancer detection using images. Rajinikanth et al. [1] developed Visual Geometry Group (VGG) + DenseNet (DN) with K-nearest neighbor (KNN) to accurately classify renal CT images from healthy cancer classes. This model effectively removed the artefacts from the CT slices using a threshold filter-supported pre-processing method to obtain high performance. This approach produced high recognition accuracy with minimal computational complexity, but it required manual verification of the threshold while implementing the threshold filter. Mahmud et al. [7] designed a DenseNet201-based approach with auxiliary losses (DenseAUXNet201) for kidney cancer classification using CT images. This approach determined the cancer stage, the tumor volume, and standard demographic features to determine suitable surgical procedures. The DenseAUXNet201 attained a minimal count of missed cases, and further, the output was not biased by the kind of cancer. Moreover, the data augmentation did not completely address the problem of data imbalance, which resulted in no significant improvement in performance. Cai et al. [18] utilized an improved version of AlexNet that incorporated fused features. Their unique approach mitigated overfitting and demonstrated impressive performance, even with smaller datasets. This research paper presents a significant advancement in deep learning applications, particularly in limited data availability. While the model has shown increased resilience to overfitting, the study did not observe a substantial rise in classification accuracy. However, this should not discourage us. The researchers propose significantly boosting classification accuracy with additional modifications or complementary techniques. Shehata et al. [19] introduced multilayer perceptron-ANN (MLP-ANN) to determine whether the renal tumor is malignant or benign. It also decided on the sub-type of malignancy to provide optimal medical management. The surface complexity between the various tumors was effectively captured using morphological features, contributing to accurately determining malignancy status. However, the MLP-ANN did not consider including demographics, like sex and age, to improve performance.

Islam et al. [5] proposed a novel approach using a shifted window (Swin) Transformer with the visual geometry group (VGG)-16 architecture. This approach reduced training time while successfully capturing low-level features and enhancing their applications’ outcomes. Integrating the Swin Transformer with VGG-16 represents a significant stride in efficiently processing complex visual data. Despite these advancements, the approach required high computational resources and involved many parameters. This high computational potential limitation, especially for deployment in environments with limited processing capabilities, points towards an area for future optimization. Patro et al. [16] introduced the deep kronecker neural network (DKN) model, exhibiting a noteworthy capacity to avoid overfitting, a common issue in deep learning models. The DKN was particularly effective in detecting even minuscule kidney stones, marking a substantial advancement in medical imaging. However, the efficiency of this approach was limited by the poor quality of the input images. The dependency on high-quality imaging indicates that inconsistency in imaging quality could affect the model’s performance, emphasizing the need for consistent, high-quality input data for optimal results. Badawy et al. [8] developed the dualistic renal disease classification (DRDC) framework. This reliable system can produce promising outcomes even with limited data, making it useful in data-scarce situations. However, the DRDC framework did not explore the possibility of enhancing classifier performance by tuning classifier parameters with various optimization techniques. This gap suggests a potential area for future research where applying optimization techniques and hybrid models could improve the model’s accuracy and efficiency.

Bhandari et al. [3] designed a lightweight CNN scheme to find abnormalities in the kidney, such as tumors, stones, and cysts. This method required minimal parameters and produced consistent results even with non-uniform sample distributions. However, this method was evaluated with limited CT images, which affected its performance. The issues endured by the available techniques for kidney cancer detection using images are as follows: VGG + DN with kNN proposed in [1], which required an artifact removal technique, and no image enhancement approach was considered to augment the accuracy of disease detection. The generalization of the dataset limited the DenseAUXNet201 [7], as the dataset did not encompass all the information required to make the classifier robust. Further, the method should have considered developing a balanced dataset with numerous diverse cases for the approach to apply to a large population. In [19], DKN was a method that effectively detected kidney stones. However, the method suffered from high computational overhead, and no optimization technique was considered to reduce the computational effort. The DRDC framework developed in [8] achieved high classification accuracy with minimum misclassifications. Still, it was futile to consider combining various classifiers to improve detection efficiency and deploying the approach on the smartphone to make it more accessible. Several deep-learning techniques were proposed for classifying kidney diseases. However, these methods required high computational effort to produce superior performances. Moreover, insufficient data to train these networks affects the classification accuracy.

## SpinalZFNet for Kidney Disease Classification Using CT Images

This research presents a hybrid DL model SpinalZFNet designed to classify kidney diseases from CT images. Initially, the input is acquired from the dataset and forwarded to the median filter. [20], which pre-processes the input images to denoise the input CT image. Later, kidney area segmentation is carried out with the help of the efficient neural network (ENet) [21] from the background image. Image augmentation is performed by padding, rotation, and translation to increase the image samples, thereby avoiding overfitting. This process is followed by feature extraction, where shape features, like area, solidity, eccentricity, perimeter, and primary axis length, as statistical features, such as mean ($\mu$), entropy (*H*), correlation (${C}_{\text{r}}$), and contrast ${C}_{\text{t}}$ [22], speeded up robust features (SURF) [23], weber local descriptor (WLD) [24] based discrete wavelet transform (DWT) [25] with Histogram of Oriented Gradients (HOG) [26] extricated. Finally, kidney disease classification into (i) Normal, (ii) Cyst, (iii) Tumor, and (iv) Stone is accomplished based on hybrid SpinalZFNet, which is the combination of SpinalNet [27] and ZFNet [28], where layers are modified.

### Dataset Description

The CT Kidney dataset [5] is collected from patients who have already been diagnosed with kidney stones, normal, cyst, or tumor findings in Dhaka, Bangladesh, by picture archiving and communication system (PACS). The contrast and non-contrast studies are performed for the urogram and abdomen to ensure a more in-depth capture of kidney details. The finalized dataset contains 12,446 distinct images. A breakdown of images includes 3709 images of cysts, 5077 of normal kidneys, 1377 of kidney stones, and 2283 showcasing tumors.

### Image Acquisition

The classification of kidney diseases is performed initially by acquiring CT images from the dataset.

1 $$K=\{{K}_{1},{K}_{2},{K}_{3},{K}_{4},\dots ,{K}_{l}\}$$

Here are the CT images provided as input, which are donated as ${K}_{l}$.

### Image Pre-Processing

The naturally available noises in the input CT image ${K}_{l}$ are effectively removed by pre-processing using a median filter [20]. It is a nonlinear method that helps preserve useful information and remove noises in CT images by considering statistics. During pre-processing using a median filter, the actual pixel grey value is converted into grey values of pixels. The median is calculated by sorting all the pixel values of the neighborhood window into numerical order and replacing the pixel considered with the middle (median) pixel value. The median is calculated by the expression as follows:

2 $${P}_{l}\left(u,v\right)={K}_{l}\left(k,l\right)$$

Here, the input CT image given to the median filter is signified as ${K}_{l}\left(k,l\right)$, and ${P}_{l}\left(u,v\right)$ denotes the output CT image obtained while pre-processing using the median filter, which is further fed into ENet for image segmentation.

### Kidney Area Segmentation Using Enet

The pre-processed CT image ${P}_{l}$ is segmented from the kidney area from the background images using Enet [21]. The Enet is highly utilized for performing low latency operations, which also perform efficiently and require fewer parameters. In general, the view of Enet is constructed on a highly efficient convolutional network architecture tailored for rapid processing without compromising accuracy. Furthermore, Enet integrates bottleneck modules to minimize computational costs while maintaining robustness in segmentation tasks. Moreover, if the bottleneck is downsampling, the bottleneck modules and the max pooling to the main branch are considered. The activations are zero-padded to match the total feature maps. The model performs full, regular, or dilated convolutions with filters, but sometimes, the convolutional operations are replaced with asymmetric convolutions.

A single block is presented at the initial stage of ENet, where the bottleneck blocks are shown in stage 1. Moreover, stages 2 and 3 have the same structure, but downsampling the input is not performed in stage 3. Stages 4 and 5 belong to the decoder, whereas the other initial three stages are encoders. The overall memory operations and kernel calls are reduced by not utilizing bias terms in any projections. Also, batch normalization is performed among each non-linear convolutional layer. In the decoding phase, max pooling is substituted with max unpooling, while padding gives way to spatial convolution devoid of bias. Pooling indices were not employed in the ultimate upsampling module due to the initial block processing of the input frame’s three channels, in contrast to the final output featuring *C* feature maps representing object categories. Moreover, due to efficiency considerations, we opted to integrate only a singular full convolution at the network’s end. This alone consumes a significant part of the decoder’s processing duration. ENet’s advanced features are essential for effectively segmenting the pre-processed CT images ${P}_{l}$.

### Image Augmentation

The segmented CT image output ${S}_{o}$ obtained using ENet allows image augmentation to avoid overfitting issues and increase diversity by randomly transforming the image samples. The different image augmentation approaches [5], like padding, rotation, and transformation, are performed in segmented CT image output ${S}_{\text{o}}$. The process carried out during augmentation is briefly enumerated further in detail. First, the padding adds pixels to the sides of the segmented CT image${S}_{o}$. It is the total number of pixels added to the segmented image while processing among kernels of CNN, where the resultant padding augmented image is represented as${W}_{o}$. Secondly, the rotation augments the segmented CT image ${S}_{o}$ by altering its orientation. This involves pivoting the image around its center to analyze the direction of the image along the angles between ${1}^{^\circ }$ and${359}^{^\circ }$. The term ${W}_{l}$ represents the augmented CT image output obtained during rotation. Translation is applied for the movement of objects available in the segmented CT image ${S}_{o}$ from one position to another. The images are preserved or randomized by translating them into black or white along $X$ and $Y$ direction or towards $X$ or $Y$ direction at the same time, where, during translation, the position bias is adjusted towards the up, down, right, and left directions. The resultant translation augmented CT image is represented as$W$. Therefore, $W=[{W}_{1},{W}_{2}, {W}_{3},\dots {,W}_{n}]$ is the final augmented CT image obtained during the augmentation of the segmented CT image${S}_{o}$.

### Features Extraction

Advanced methods are used to extract pertinent information from the images during feature extraction. Specifically, features such as mean ($\mu$), entropy (*H*), correlation (${C}_{\text{r}}$), and contrast ${C}_{\text{t}}$ [22], SURF [23], WLD [29] based DWT [25] with HOG [26] are used for extraction of features from augmented CT images $W$. The extraction process performed is explained below.

#### SURF

The augmented CT image $W$ is fed into SURF to extract textural features. The SURF is a local feature commonly used to locate interesting points and determine descriptors.

Localization of interest point: in general, the SURF detector is based on the Hessian matrix:

3 $${\varvec{H}}\left(Z,\sigma \right)=\left[\begin{array}{cc}{L}_{xx}\left(Z,\sigma \right)& {L}_{xy}\left(Z,\sigma \right)\\ {L}_{xy}\left(Z,\sigma \right)& {L}_{yy}\left(Z,\sigma \right)\end{array}\right]$$

where, ${L}_{xx}(Z, \sigma )$ is the convolution of Gaussian second order of derivative in point $Z$, which is also similar for ${L}_{xy}(Z, \sigma )$ and ${L}_{yy}(Z, \sigma )$. The Hessian is determined for selecting interest point scale and location, whereas non-maximum suppression is applied for localizing the interest points in image and scale space. Moreover, the interpolation of image and scale space is performed finally to identify the local maxima of the appropriate Hessian matrix.

Descriptor interest point: in this phase, a unique orientation is assigned to the former, and image rotation is performed by gaining invariance by constructing a circular region around the detected point of interest. The orientation is computed using Haar wavelet responses in both $x$ and $y$. The Haar wavelets are quickly computed via integral images, similar to the Gaussian second-order approximated box filters. The dominant orientation is estimated and included in the interest point information. In the next step, square regions are extracted around the point of interest for constructing the SURF descriptor. The spatial data is retained by splitting the windows into sub-regions, where Haar wavelets are extracted in each sub-region. The polarity information of image intensity changes is obtained by summing the absolute values. Therefore, the vector $V$ used to underline the intensity patterns of the sub-regions is given in the following expression:

4 $$V = \left[ {\sum d_{x} , \sum d_{y} ,\sum \left| {d_{x} } \right|,\sum \left| {d_{y} } \right| } \right]$$

where, ${d}_{x}$ and ${d}_{y}$ signifies the wavelet response over horizontal and vertical directions. The Haar wavelets are invariant to contrast and are also invariant to illumination bias while normalizing the resultant descriptor vector, and the extracted SURF feature is signified as ${J}_{\text{l}}.$

#### WLD-Based DWT with HOG

The discrete wavelet transform (DWT) refers to linear transformation, a function of the data vector, the length of which is associated energy. In the wavelet transformation, two steps are used to isolate the function of different natural image subbands and isolate image wavelet coefficients. The expression used to compute the mean value of DWT coefficients is given in Eq. (5):

5 $${\mu }_{t}=\text{mean} (\text{DWT})$$

where, the determined inference value from the initial value is represented as ${\mu }_{t}$. The DWT splits the augmented CT image $W$ into three subbands, as Low-Low (LL) ${\phi }_{1}$, Low‐High (LH) ${\phi }_{2}$, and High‐High (HH) ${\phi }_{3}$. The split subbands are separately fed into WLD for further augmented image extraction. Further, the WLD used a simple yet robust local texture descriptor for digital images, which Weber’s Law inspired. It is composed of two components: differential excitation and orientation. The differential excitation of the present pixel is determined using the expression given below:

6 $$g\left({f}_{h}\right)=\text{arctan}\left[\sum_{i=0}^{t-1}\frac{{f}_{i}-{f}_{h}}{{f}_{h}}\right]$$

Here, ${f}_{h}$ signifies the present pixel. ${f}_{i}$ represents the *i*-th neighboring pixel of ${f}_{h}$ and the total amount of neighbor are represented as $t$. Moreover, the present pixels’ gradient orientation component is determined by Eq. (7):

7 $$\phi \left({f}_{h}\right)=\text{arctan}\left[\frac{{\phi }^{(11)}}{{\phi }^{(10)}}\right]$$

where the output obtained from the two filters is assumed as ${\phi }^{(11)}$ and ${\phi }^{(10)}$. The subbands are obtained from DWT by filtering the image using the kernels depicted as follows:

8 $$\it Y_{{\text{o}}} = \left( { - 1, 0, 1} \right)\,{\text{and}}\,\it Y_{{\text{s}}} = \left( {\begin{array}{*{20}c} { - 1} \\ 0 \\ 1 \\ \end{array} } \right)$$

Also, the orientation $w(p,q)$ as well magnitude $v(p,q)$ is determined using the expression using the Eq. (9) as follows:

9 $$w\,(p,q) = \sqrt {(A_{x}^{2} + A_{y}^{2} )} \,{\text{and}}\,v\,(p,q) = A_{y} /A_{x}$$

Here, the vertical and horizontal gradients are represented as ${A}_{y}$ and ${A}_{x}$, and the final extracted feature obtained by WLD-based DWT with HOG feature extractor is signified as ${J}_{2}$. Moreover, the extracted textural features are given by ${\varvec{J}}= [{J}_{1}, {J}_{2}]$, which further allows the extraction of the most significant vector features using shape and statistical feature extractors.

#### Shape Features

Shape features, like area, perimeter, major axis length, minor axis length, and convex hull, are used to calculate the shape and find its value. Details of shape features are provided as follows: The shape is extracted using compactness, which is calculated by using the ratio of perimeter, and the extracted area feature is signified as ${H}_{1}$. The extent to which the shape is concave or convex is determined using solidity [30], which is computed by:

10 $${H}_{2}=\frac{\alpha }{\beta }$$

where the object area is represented as $\alpha$, the convex hull is an outside line that covers all the points and is signified as $\beta$, and the extracted solidity feature is signified as ${H}_{2}$. Eccentricity is the calculation of the length of the central axis as a proposition to the length of the minor axis [30], given by expression as:

11 $${H}_{3}= \frac{\zeta }{\delta }$$

Here, the extracted eccentricity feature is symbolized as ${H}_{3}$, the minor axis is signified as $\zeta$ and the major axis is denoted as $\delta$. Perimeter is the summation of pixels at all the sides of an image’s shape. This distance, represented by Eq. (12), measures the shape’s size in the respective dimension:

12 $${H}_{4}= \sqrt{{({a}_{2}{-a}_{1})}^{2}+{({b}_{2}{-b}_{1})}^{2}}$$

where, the endpoints are represented as $\left({a}_{1},{b}_{1}\right)$ and ${(a}_{2},{b}_{2})$ and the extracted major axis length is signified as ${H}_{4}$. Thus, the extracted shape features are finally given by the expression: ${H}_{\text{shape}}=\{{{H}_{1},{H}_{2},H}_{3},{H}_{4}\}$.

#### Statistical Features

The different statistical feature extractors, like mean, entropy, correlation, and contrast are extracted from the augmented CT images. The extraction process performed is briefly described as follows: The pixels available in the image under a certain dimension are termed mean [5], which is computed using:

13 $${H}_{5}= \frac{1}{R}\sum_{c=i}^{R}{I}_{c}$$

Here, the image pixel is signified as $I$, and ${H}_{5}$ indicates the extracted mean feature. The pixel with high information is determined using entropy [5]. The formula is expressed as follows:

14 $${H}_{6}=\sum_{c,d}p\left(c,d\right)\text{log} p(c,d)$$

$p\left(c,d\right)$ is the probability of occurrence of pixel value $c,d$ in the image and ${H}_{6}$ signifies the extracted entropy feature. The correlation [22] is used to describe the correlation of a pixel with another pixel of the image, where the expression gives the correlation feature:

15 $${H}_{7}=\sum_{c,d}\frac{(c-{\Psi }_{c})(d-{\Psi }_{d}){\eta }_{c,d}}{{\mathcal{L}}_{c}{\mathcal{L}}_{d}}$$

where, $c$ and $d$ indicates section, $\Psi$ signifies mean, the standard deviation is represented as $\mathcal{L}$, the Grey-Level Co-occurrence Matrix (GLCM) is symbolized as $\eta$, and the extracted correlation feature is given by ${H}_{7}$. Contrast is the extent reduced level arrangement of a neighborhood in the image and is given in Eq. (16) as

16 $${H}_{8}=\sum_{c,d}{\left|c-d\right|}^{2}{\eta }_{c,d}$$

where the extracted contrast feature is signified as ${H}_{8}$. Here ${H}_{\text{stat}}=\{{H}_{5},{{H}_{6},{H}_{7},H}_{8}\}$ is the final extracted shape given in the expression. Finally, all features are extracted further to classify the kidney disease.

### Kidney Disease Classification Using SpinalZFNet

**Fig. 1:**
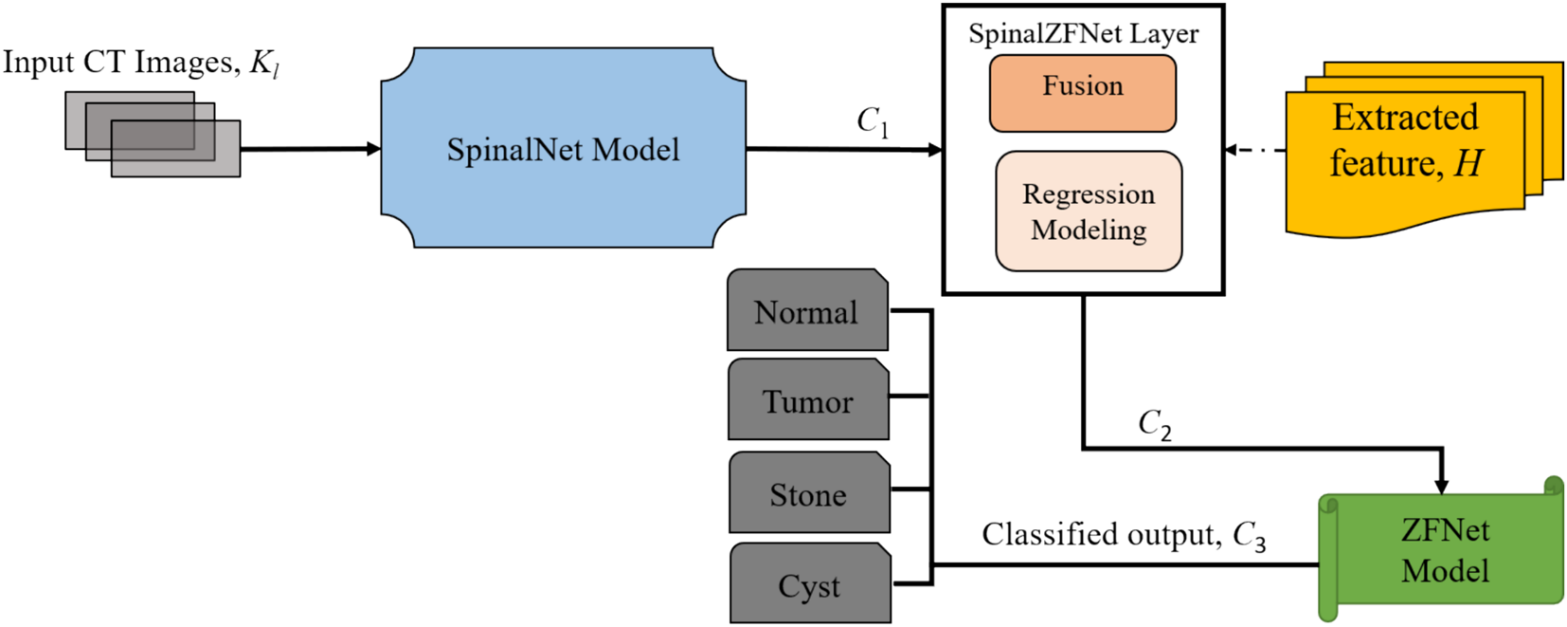
Block diagram of SpinalZFNet used for kidney disease classification

The abnormalities available in the kidney can potentially cause an adverse effect on health. Hence, it is necessary to detect the exact kidney disease to provide accurate treatment for kidney disease. Thus, the SpinalZFNet model is designed in this research to precisely classify kidney disease into normal, cyst, tumor, and stone to provide early diagnosis. Here, the SpinalZFNet model is initially processed by the SpinalNet model, which is trained to recognize complex patterns associated with kidney conditions. This preliminary stage acts as a filter, refining the input and preparing it for more detailed analysis. Then, the extracted feature $H$ along with the output obtained from SpinalNet ${C}_{1}$ is sent to SpinalZFNet layer to obtain ${C}_{2}$. This layer facilitates the fusion of technologies from SpinalNet [27] and ZFNet [28], employing a fully connected (FC) [31] setup for regression modelling, which aims to identify relationships among the inputs and perform the fusion. This FC setup is particularly well-suited for tasks involving various integral and derivative calculations due to its dense network of node connections. It effectively integrates and extracts high-level features from the data, enhancing the model’s capability to handle complex information processing. Consequently, the output ${C}_{2}$ obtained from the SpinalZFNet layer is sent to the ZFNet model for the final kidney disease classification. The final classified output of ${C}_{3}$ is obtained, which is classified as (i) Normal, (ii) Cyst, (iii) Tumor, and (iv) Stone by forwarding the output ${C}_{2}$ obtained from SpinalZFNet layer to ZFNet model. The ZFNet model component improved the model’s flexibility, maintainability, and potential for fine-tuning. The ZFNet model is a pre-trained feature extraction module easily integrated into the SpinalNet model, using transfer learning principles to enhance the model’s performance. The block diagram of SpinalZFNet used for kidney disease classification is displayed in Fig. 1.

#### SpinalNet Model

The SpinalNet model [27] is designed by considering the function of the human spinal cord, where the input ${K}_{l}$ CT image are given to the SpinalNet continuously in a step-by-step format. The global and local output sends a modulated input version pass through each SpinalNet layer. The model structure contains input sub-layer, intermediate sub-layer, and output layer. The input is split and forwarded to multiple hidden layers known as intermediate network layers. The intermediate split layer of SpinalNet generally acquires two components, one from the previous intermediary layer split outcome and one from the present input split outcome. The weighted outputs of intermediate splits are passed and added with each layer’s output split. The hidden layers of intermediate splits comprise two neurons, where the expression gives the output of the neuron in the hidden layer,

17 $${C}_{1}=\sum_{i=1}^{N}{(w}_{i}){q}_{i}+{b}_{i}$$

**Fig. 2:**
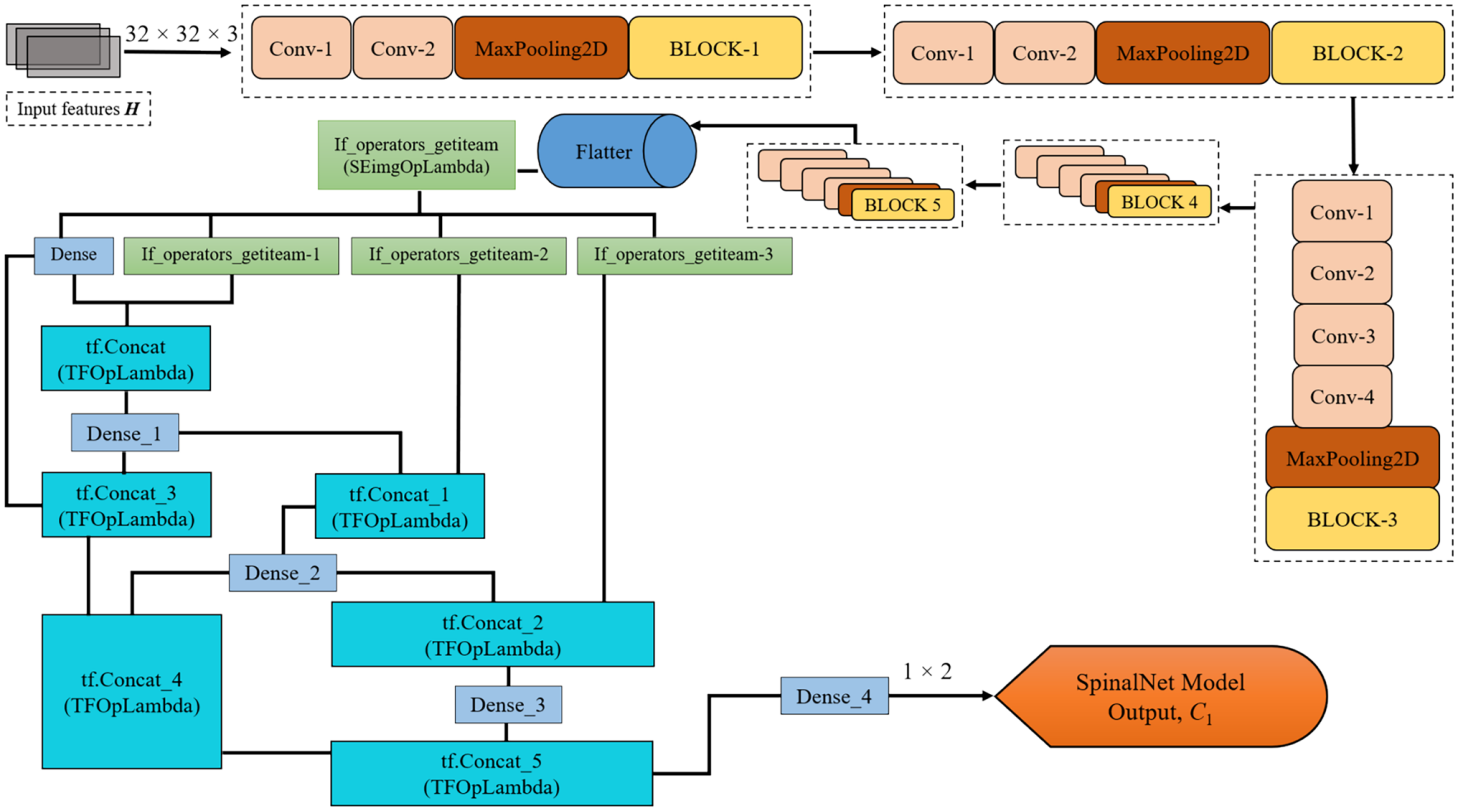
Structure of SpinalNet Model

Here, the threshold value of ${b}_{i}$ represents the bias associated with the neuron in the hidden layer. This bias helps adjust the output and the weighted sum of the inputs to the neuron. The weights ${w}_{i}$ correspond to the significance of each input ${q}_{i}$ and the summation runs over all $N$ inputs to that neuron. The depth and architecture of the SpinalNet model enable it to effectively identify intricate patterns and relationships within the input data, making it particularly suited for identification of kidney diseases once the input CT image is fed into the SpinalNet model. The structure of SpinalNet model is depicted in Fig. 2. The model integrates the outputs from various stages of the network, including the convolutional blocks (Conv-1, Conv-2, MaxPooling2D, BLOCK-1 to BLOCK-5) and the dense operators (Dense_1 to Dense_4). The convolutional blocks extract local features from the input data, while the dense operators process the flattened output from the convolutional blocks. The concatenation operations (tf.Concat_1 to tf.Concat_5) combine these feature representations at different levels, allowing the SpinalNet model to leverage multi-scale information.

#### SpinalZFNet Layer

The resultant output ${C}_{1}$ obtained from the SpinalNet model and the extracted features ${\varvec{H}}$ is fed into SpinalZFNet layer for the fusion of SpinalNet [27] and ZFNet [28]. The FC [31] is used for regression modeling in SpinalZFNet layer to perform fusion by determining the relationship between the given inputs. FC generally provides solutions to various integral and derivative tasks by establishing dense node connections. These layers integrate information, capturing high-level features from the input data. In the context of the provided equation, the fusion computes the weighted sum of features from different intervals or sources. Layers at *p*-th interval leverage this weighted approach to better capture the intricacies and patterns across multiple data points. Specifically, $p$ is given in Eq. (18),

18 $$p= \sum_{I=1}^{2}\sum_{F=1}^{s}\sum_{G=1}^{t}{H}_{IFG}\times {X}_{IFG}$$

Here, the available total amount of features is indicated as $t, s$ indicates total available data, the weight coefficient is signified as $X$. At (*p* – 1)-th interval the expression gives the SpinalZFNet layer output,

19 $${p}_{1}=\sum_{E=1}^{4}{H}_{ST}\times {X}_{E}$$

where, the statistical feature is represented as ${H}_{ST}$. Moreover, the output of SpinalZFNet layer at (*p*-2)-th interval is designated as,

20 $${p}_{2}=\sum_{E=1}^{4}{H}_{SN}\times {X}_{E}$$

where ${H}_{SN}$ signifies shape.

21 $$U=f(p,{p}_{1},{p}_{2})$$

Equation (21) is given by

22 $${C}_{2}=U\times p+\frac{1}{2}U\times {p}_{1}+\frac{1}{6}\left(1-U\right)\times {p}_{2}+\frac{1}{24}\left(1-U\right)\left(2-U\right)\times {C}_{1}$$

Thus, applying the value of $p,{p}_{1}$ and ${p}_{2}$, Eq. (22) becomes.

23 $${C}_{2}= U\times \left[\sum_{I=1}^{2}\sum_{F=1}^{s}\sum_{G=1}^{t}{H}_{IFG}\times {X}_{IFG}\right]+\frac{1}{2}U\times \left[\sum_{E=1}^{4}{H}_{ST}\times {X}_{E}\right]+\frac{1}{6}\left(1-U\right)\left[\sum_{E=1}^{4}{H}_{SN}\times {X}_{E}\right]+\frac{1}{24}\left(1-U\right)\left(2-U\right)\times {C}_{1}$$

Equation (23) is the SpinalZFNet layer’s final resultant output, which is forwarded to the ZFNet model for kidney disease classification.

#### ZFNet Model

A type of CNN, the ZFNet model [28, 32] is introduced by visualizing the intermediate feature maps. The ZFNet structure consists of convolutional and fully connected (FC) layers, where the output is fed into the subsequent layer for further processing and refinement. This hierarchical structure ensures a progressive refinement of features as the data traverses through the network. The kernel map of the second layer is connected to the kernel of the third convolutional layer, and the fully connected layer neurons are connected to the neurons of the preceding layer. In addition, next to the first and second convolutional layers, the response-normalization layer is connected. Followed by the response-normalization layer and the fifth convolution, the max pooling layer is connected. Rectified Linear Units (ReLU) non-linearity is applied to the convolutional and fully connected layer output. The kidney disease classification process carried out in the ZFNet model is explained below.

The output obtained from the SpinalZFNet layer, ${C}_{2}$, is fed initially into a convolutional layer that forwards the output in a successive or sequential form via interconnected convolutional layers. Here, the input is convolved with kernels for the creation of feature maps, and the convolution operation performed in a convolutional layer is designated as:

24 $$\varpi = {C}_{2}\vartheta$$

From the expression, the

25 $$L=t(\varpi )= {t(C}_{2}\vartheta )$$

The activation function is signified as $t$, and the matrix is represented as $L$. The down-sampling of feature maps available in the channels is performed in the max-pooling layer. Moreover, the summation is performed for the determination of main features, and finally, a stride $J\times J$ is used to generate a reduced subset after the output obtained from the convolutional layer is fed into the max pooling layer, which is expressed as

$$o:L\to \theta$$

26 $${\text{R}}^{{f}^{2}\times {f}_{a}}\to {\text{R}}^{\frac{{f}^{2}}{{J}^{2}}\times {f}_{a}}$$

Here, the reduced subset is signified as $\theta$ and ${f}_{a}$ indicates the pooling operation carried out in channels. The max-pooling operation is performed by considering the highest pooling value and is designated as

27 $$\theta ={B}_{\text{max}}\left(t\left({C}_{2}\vartheta \right)\right)$$

Here, the max-pooling function is represented as ${B}_{\text{max}}$ and is formulated as

28 $${B}_{\text{max}}= {\Gamma }_{\text{max}}(O)O$$

where the identity matrix is signified as ${\Gamma }_{\text{max}}\left(O\right)\epsilon {\text{R}}^{\frac{{f}^{2}}{{J}^{2}}\times {f}^{2} }$

In the ZFNet model, the flattening layer is used for the conversion of feature maps into a set of elements before forwarding it to the fully connected layers

$$z=\left[{v}_{1}, {v}_{2},\dots {,v}_{f}\right]$$

29 $${{=[\Gamma }_{1}v}_{1}, {{\Gamma }_{2}v}_{2},\dots ,{{\Gamma }_{f}v}_{f}]$$

where, the *f*-th max pooling layer output of the pooling layer and the flattening operation executed in the flattened layer is given in Eq. (30).

30 $${\varvec{z}}=F\left[{\varvec{z}}\right]= \left[\begin{array}{c}\begin{array}{c}{v}_{1}\\ {v}_{2}\\ \vdots \\ {v}_{f}\end{array}\end{array}\right]= \left[\begin{array}{c}{{\Gamma }_{1}v}_{1}\\ {\Gamma }_{2}{v}_{2}\\ \vdots \\ {{\Gamma }_{f}v}_{f}\end{array}\right]$$

The dense layer performs linear operation by determining the relationship between the inputs. Thus, the resulting output is designated as

31 $$z = F\left[ z \right] = F\Gamma_{n} N_{n} r\left( {C_{2} \vartheta_{n} } \right)$$

The expression gives the final output of CNN,

32 $${C}_{3}={G}_{k}\left({D}_{k}\dots {G}_{2}{(D}_{2}{G}_{1}\left({(D}_{1}G\right)\right)\dots )$$

The weight and activation functions are denoted as ${D}_{k}$ and ${G}_{k}$ at the *k*-th FC layer, respectively. Activation function output is donated as

33 $${{E}_{3}C}_{3}=E\left({D}_{k}\dots {G}_{1}{(D}_{1 }\times {{\Gamma }_{2}N}_{2}[{{\Gamma }_{1}N}_{1} ({{C}_{2}^{{f}_{1}}\vartheta }_{1}){]}^{{f}_{2}} {\vartheta }_{2})\dots {\vartheta }_{n}\right)$$

**Fig. 3:**
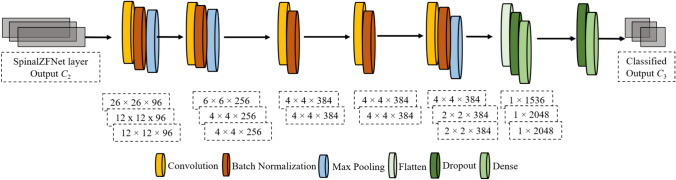
Structure of ZFNet model

The activation function is indicated ${{E}_{3}C}_{3}$ The structure of the output of the ZFNet model is presented in Fig. 3.

## Result

This section discusses the results obtained by applying the SpinalZFNet model to classify kidney diseases. Our goal was to leverage the advantages of the spinal architecture and the ZFNet model to achieve a more precise and comprehensive classification. We conducted experiments that demonstrated its effectiveness. The SpinalZFNet was designed to classify kidney disease using the CT kidney dataset [5].

### Experimental Image Results

**Fig. 4:**
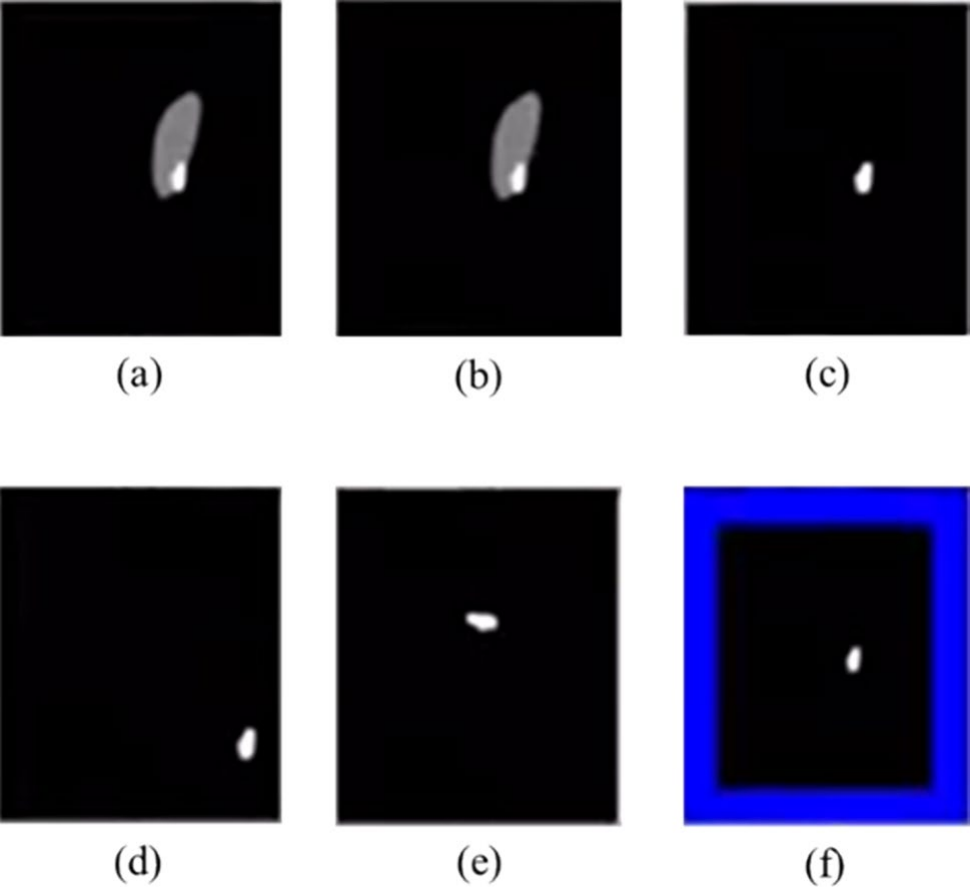
Experimental image outcomes. **a** Input, **b** pre-processed, **c** segmented, **d** translation, **e** rotation, and **f** padding augmented CT image

The resultant images recorded by SpinalZFNet during kidney disease classification display in Fig. 4. The input CT image show in Fig. 4a, b depicts the pre-processed CT image, and Fig. 4c gives the segmented CT image. Moreover, Fig. 4d–f portray the translation, rotation, and padding augmented CT image. Given the dataset, such as kidney disease images, calculate the number of features each method extracts from a single image as follows:

(1) Speeded-up robust features (SURF): the image is of a kidney disease segment image based on a black background. SURF detects only a few key points due to the high contrast and small area of interest. SURF detects five key points. With a descriptor length of 64, that would be 320 features (5 key points $64\times 64$ descriptor length). (2) WLD-based DWT with HOG (Wavelet-Local Binary Patterns with Histogram of Oriented Gradients): after applying DWT, approximation coefficients (cA) and detail images (cH, cV, cD) are all considered for HOG feature extraction. With an HOG descriptor window size of $64\times 64$ pixels, $8\times 8$ cells, and 9 histogram bins per cell, get a feature vector of length $7 \times 7 \times 9 = 441$ from a single channel (due to the downscaling effect of DWT). (3) Shape features: given that the image’s shape is simple, calculate one value each for area, perimeter, major axis length, minor axis length, and convex hull. This results in 5 shape features. (4) Statistical features: the image’s region has some statistical measures: mean intensity, entropy (calculated from the histogram of pixel intensities), correlation, and contrast (from a gray-level co-occurrence matrix, (GLCM)). This extracts 4 statistical features.

### Training and Implementation

This study trained the proposed SpinalZFNet model using 100 epochs on the Google Colab platform. The training was conducted on a single 12 GB NVIDIA Tesla K80 GPU with a maximum continuous usage limit of 12 h. The GPU mode was utilized for faster execution. However, the completion time of the training process depended on factors such as network speed and dataset size.

Table 1 presents a comprehensive overview of the hyperparameters used in the SpinalZFNet model. We employed random hyperparameters to train the proposed model. We utilized multiple optimizers such as Adam, SGD, and RMSProp for the random hyperparameters. We set the default learning rate to 0.001. During the training, we incorporated critical features such as a reduced learning rate, model checkpoint, and early stopping. The reduced learning rate helped adjust the learning rate dynamically based on the training progress. The monitoring of the validation accuracy was conducted, and if it did not exhibit improvement for five consecutive epochs, with a minimum change of 0.0001, the present learning rate was halved. This process continued until the last epoch.

**Table 1:** Details of the hyperparameters used in the SpinalZFNet

Hyperparameter	Variables	Randomly used
Optimizer	Adam, Nadam, SGD, RMSProp	Adam, SGD, RMSProp
Batch size	12, 16, 32, 64	32
Epochs	[55, 65, 100, 120, 150]	100
Learning rate	0.01, 0.001, 0.0001	Learning rate adjusted based on the training progress

We also utilized the model checkpoint callback to store the weights of the top-performing model, which was determined by its validation accuracy. Early stopping was employed to ascertain the optimal number of epochs required for the training process. After 50 epochs, if no performance improvement was observed, the training process was stopped to avoid overfitting. The batch size, another essential hyperparameter, was set to 32 in the SpinalZFNet model, as it yielded satisfactory results. Larger batch sizes allow for better parallelization, which may negatively affect the model’s generalization ability. The study employed the SoftMax cross-entropy loss function, which is well-suited for addressing multiclass classification problems—quantifying the discrepancy between the output of the network and its corresponding labels.

**Fig. 5:**
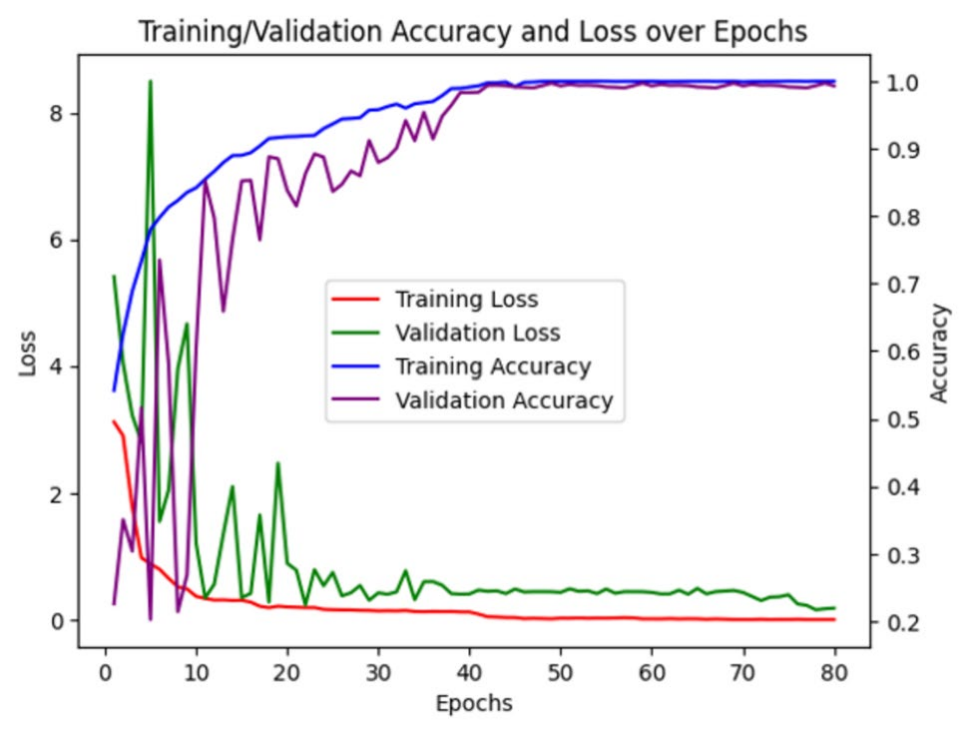
Training and validation accuracy and loss curves on the CT images of the SpinalZFNet model

Figure 5 presents the learning curves of the SpinalZFNet model on the CT dataset. The curves for training and validation, as well as the loss curves, show an effective convergence across the dataset. The training process was stopped after 80 epochs to avoid overfitting, as no improvement in performance was observed for five consecutive epochs. The optimal number of epochs was utilized for training. This means that the data used for training the model is a good representation of the data used for validation. The fact that the training and validation curves are close to each other suggests that the model is reliable when tested. This provides evidence that the SpinalZFNet model is not overfitting.

### Performance Metrics

Various performance metrics [33, 34] are utilized to identify SpinalZFNet’s superiority, including the classification performance, which are explained below.

Accuracy: is the relationship between the original and expected outcome obtained by SpinalZFNet during kidney disease conditions.

34 $$\text{Accuracy}=\frac{{n}_{\text{TP}}+ {n}_{\text{TN}}}{{{n}_{\text{FP}}+{n}_{\text{FN}}+n}_{\text{TP}}+{n}_{\text{TN}}}$$

where ${n}_{\text{TP}},{n}_{\text{TN}}, {n}_{\text{FP}},{n}_{\text{FN}}$ represent the number of true positives, true negatives, false positives, and false negatives, respectively.

Sensitivity: the positive samples classified precisely by SpinalZFNet from the input total positive samples is called sensitivity, where the sensitivity is given by

35 $$\text{Sensitivity}=\frac{{n}_{\text{TP}}}{{n}_{\text{TP}}+{n}_{\text{FN}}}$$

Precision: the precision is defined as the positive samples falsely classified SpinalZFNet from the total positive samples and is defined as

36 $$\text{Precision}=\frac{{n}_{\text{TP}}}{{n}_{\text{TP}}+{n}_{\text{FP}}}$$

Specificity: the specificity is defined as the negative samples precisely classified by SpinalZFNet from the total input negative samples and is expressed by

37 $$\text{Specificity}= \frac{{n}_{\text{TN}}}{{n}_{\text{TN}}+{n}_{\text{FP}}}$$

F1-Score: Precision and Sensitivity are used by SpinalZFNet for F1-Score calculation.

38 $${\text{F}}1 - {\text{Score}} = 2 \times \frac{{{\text{Precision}} \times {\text{Sensitivity}}}}{{{\text{Precision}} + {\text{Sensitivity}}}}$$

### Performance Assessment

The *K*-fold value and percentage learning set are used to determine SpinalZFNet’s classification performance during kidney disease classification.

#### Analysis Using *K*-fold

**Fig. 6:**
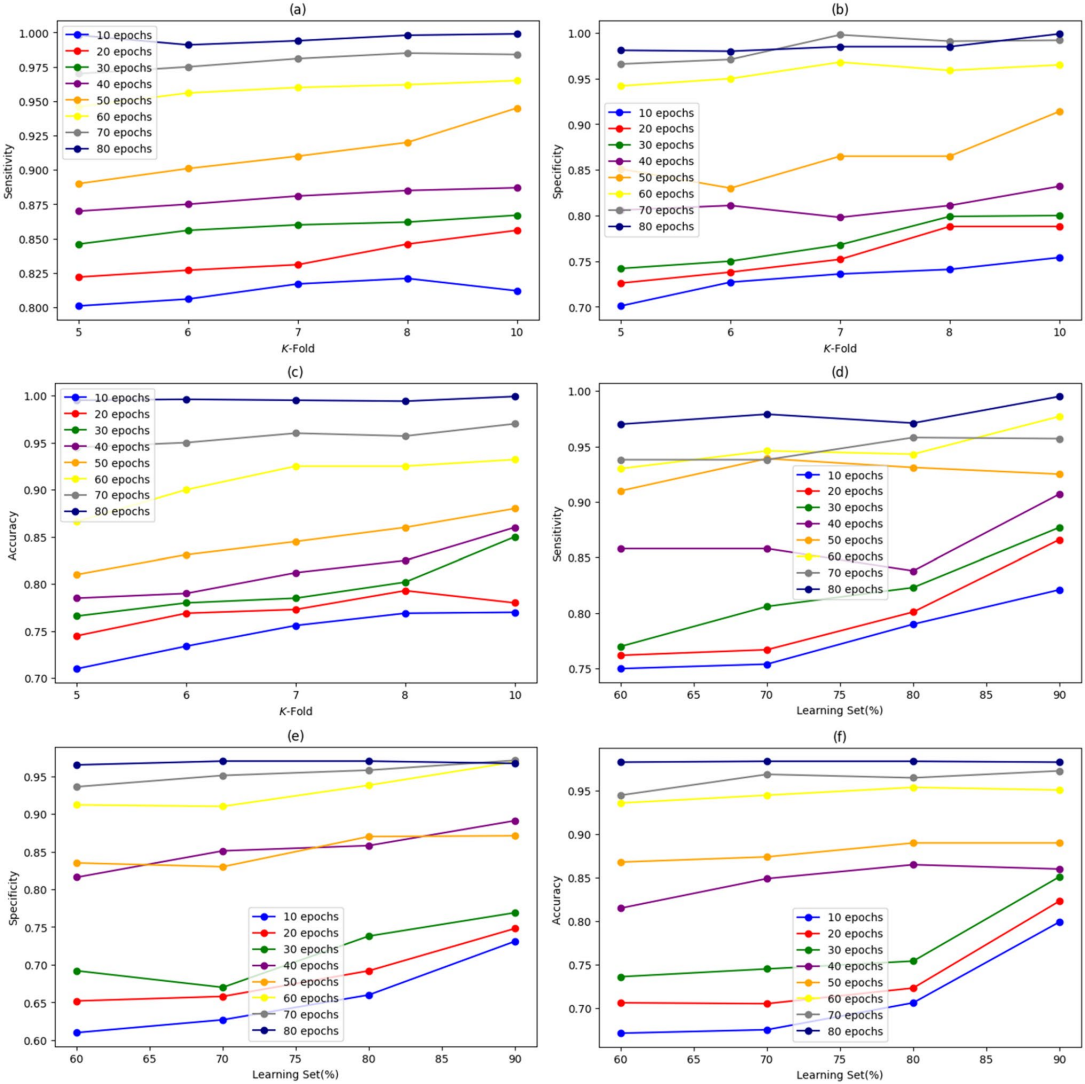
Performance analysis of SpinalZFNet using *K*-fold (**a** sensitivity, **b** specificity, **c** accuracy) and learning set, (**d** sensitivity, **e** specificity, and **f** accuracy)

The performance validation of superiority used for kidney disease classification using *K*-fold is illustrated in Fig. 6. The model’s accuracy progression over different epochs is presented in Fig. 6a. It can be observed that with the increase in epochs, there is a consistent improvement in accuracy. For a *K*-fold value 10, the recorded sensitivity for epochs 10 to 80 is 80.9%, 86.3%, 87.1%, 88.0%, and 99.0%, respectively, highlighting the model’s learning capability and stability over extended training. In Fig. 6b, the 78.1%, 79.8%, 81.9%, 83.1%, and 85.13% specificity is recorded by SpinalZFNet for *K*-fold values 10 for 10 to 80 epochs. Also, in Fig. 6c the evaluation of performance using accuracy is depicted. For *K*-fold value 10, the SpinalZFNet measured accuracy for epochs 10 to 80 is 79.9%, 81.3%, 83.1%, 85%, and 99%.

#### Analysis Using Learning Set

The analysis of the performance of SpinalZFNet utilized for the classification of kidney disease using a learning set is shown in Fig. 6. In Fig. 6d, the performance analysis using sensitivity is portrayed. For learning a set of 90%, the SpinalZFNet obtained a sensitivity of 81.2–98.3% for 10–80 epochs. Figure 6e shows that the model achieved a specificity of 75–98.1% on 10–80 epochs. Figure 6f shows the model’s performance using accuracy. The SpinalZFNet’s accuracy was measured for epochs 10 with a value of 79.5% and for 80 epochs with a value of 98.4%, respectively.

### Comparative Analysis

Traditional classification models have been used to analyze data, including VGG + DN with KNN [1], DenseAUXNet201 [7], MLP-ANN [18], and DRDC [8]. In this study, SpinalZFNet was compared to these models using the *K*-fold value and percentage of the learning set. Metrics such as sensitivity, specificity, precision, accuracy, F1-score, confusion matrix, and ROC curve were used to evaluate performance.

#### Analysis Using *K*-fold

**Fig. 7:**
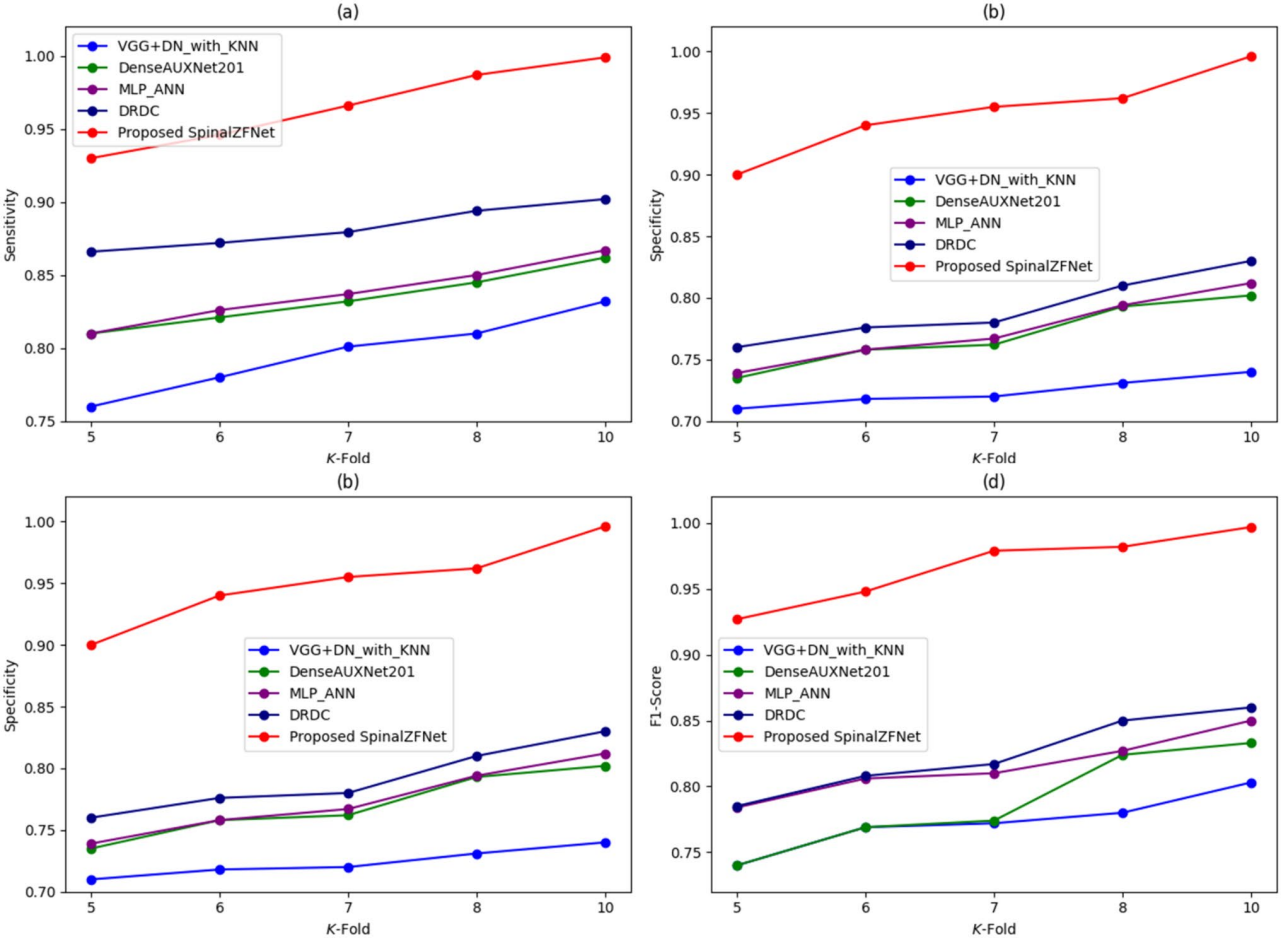
Comparative analysis of SpinalZFNet using *K*-fold: sensitivity, specificity, accuracy, and F1-score

The comparative analysis of SpinalZFNet efficiency using the *K*-fold value during kidney disease classification represents in Fig. 7. The performance analysis is based on the sensitivity, specificity, accuracy, and F1-Score of various approaches used for kidney disease classification. The sensitivity measured by SpinalZFNet is 99.9% for the *K*-fold value of 10. The classical classification approaches, like VGG + DN with KNN, DenseAUXNet201, MLP-ANN, DRDC, and proposed SpinalZFNet recorded sensitivity of 83.2%, 86.2%, 86.7%, 89.4%, and 99.9%, as shown in Fig. 7. The specificity 99.6% is notably higher in SpinalZFNet, indicating fewer false positives and more reliable performance in distinguishing non-disease cases. Precision is highest for SpinalZFNet at 99.6%, suggesting its predictions are highly trustworthy. The accuracy measured for *K*-fold value of 10 by the disease classification approaches is 80.8% by VGG + DN with KNN, 82.3% by DenseAUXNet201, 83.0% by MLP-ANN, 86.5% by DRDC, and 99.8% by SpinalZFNet. The analysis proved that SpinalZFNet attained better performance than the prevailing DRDC model. The F1-Score for *K*-fold value 10 is 78% by VGG + DN with KNN, 82.4% by DenseAUXNet201, 82.7% by MLP-ANN, 85.8% by DRDC, and 99.7% by SpinalZFNet. The analysis proved that SpinalZFNet performed better than the state-of-the-art models.

#### Analysis Using Learning Set

**Fig. 8:**
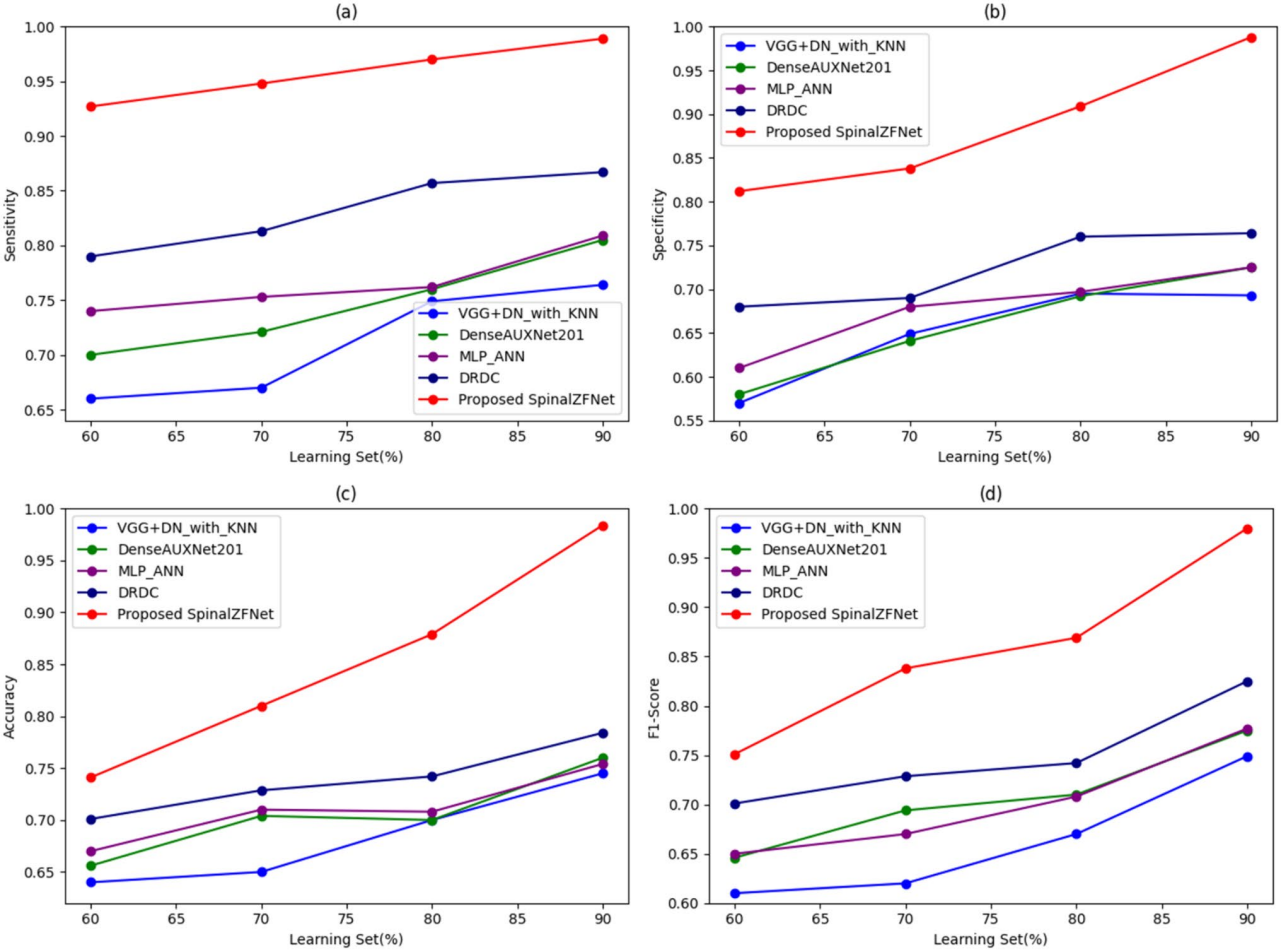
Comparative analysis of SpinalZFNet using learning set: sensitivity, specificity, accuracy, and F1-score

The validation of the performance of SpinalZFNet used for kidney disease classification by varying percentages of the learning set is displayed in Fig. 8. Figure 8 presents the evaluation of classification techniques using sensitivity, specificity, precision, accuracy, and F1-Score. The methods used for the classification of kidney disease sensitivity measured by SpinalZFNet, VGG + DN with KNN, DenseAUXNet201, MLP-ANN, and DRDC are 98.9%, 76.4%, 80.56%, 80.97%, and 86.7%, respectively. The specificity recorded for 50% of the learning set by SpinalZFNet is 98.8%. Similarly, the traditional classification techniques, such as VGG + DN with KNN, DenseAUXNet201, MLP-ANN, and DRDC, obtained specificity of 72.3%, 72.5%, 72.5%, and 76.4%. It is proven that the SpinalZFNet achieved the best performance of 97.7% compared to the traditional VGG + DN with KNN, DenseAUXNet201, MLP-ANN, and DRDC approaches, regarding precision, 73.3%, 74.6%, 74.68%, and 78.69% achieved values for classification approaches. Moreover, the analysis of kidney disease classification approaches shows that the accuracy measured by SpinalZFNet for learning set value is 98.4%, and the accuracy measured by prevailing kidney disease classification models, such as VGG + DN with KNN, DenseAUXNet201, MLP-ANN, and DRDC, is 74.5%, 76%, 70.54%, and 78.4%. Lastly, the F1-score of VGG + DN with KNN is 74.9%, DenseAUXNet201 77.5%, MLP-ANN 77.7%, DRDC 82.5%, and SpinalZFNet 98.0%. The analysis revealed that the SpinalZFNet performed better than the prevailing state-of-the-art models used for disease classification.

## Comparative Discussion

**Fig. 9:**
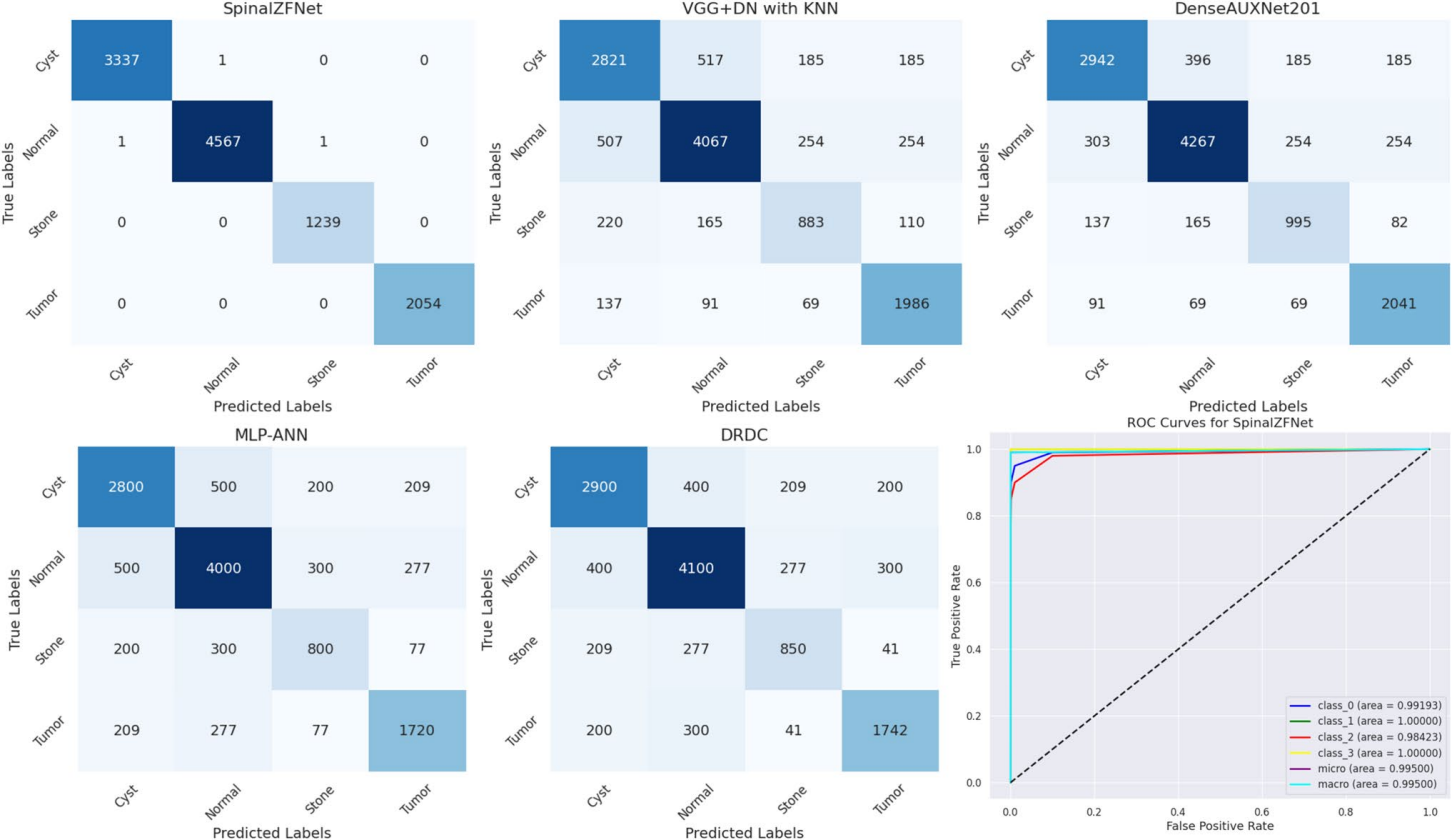
Performance analysis using confusion matrix of SpinalZFNet with state-of-the-art models and ROC curves for SpinalZFNet

The classification performance of SpinalZFNet is determined by validating the performance with prevailing techniques used for the classification of kidney disease. The results obtained by the classification approaches for different performance metrics validated using a learning set, and *K*-fold are given in Table 2. Based on experimental investigation, it is proven that the SpinalZFNet, with the learning set evaluation, emerges as the most competent model in terms of sensitivity, specificity, precision, accuracy, and F1-Score at 98.9%, 98.8%, 97.7%, 98.4%, and 98.0%. With K-Fold evaluation, the SpinalZFNet maintains the best performance with a maximum sensitivity of 99.9%, specificity of 99.6%, precision of 99.6%, accuracy of 99.8%, and F1-score of 99.7%. In contrast, during kidney disease classification, the SpinalZFNet effectively solves complexity issues using its pre-trained architecture. Similarly, it utilizes highly efficient feature maps to enhance classification accuracy by eliminating the undesired features that lead to incorrect predictions. The comparison of the provided confusion matrices for the models MLP-ANN, DRDC, SpinalZFNet, VGG + DN with KNN, and DenseAUXNet201 yields several insights into their performance in classifying the four classes: cyst, normal, stone, and tumor. SpinalZFNet stands out as the best model, particularly for the critical “Tumor” classification, where it has the highest number of true positives and lowest false negatives, making it the most suitable model among those compared for classifying the CT images of Cyst, Normal, Stone, and Tumor. SpinalZFNet model performance comparison with all models using a confusion matrix provides in Fig. 9. The ROC curve illustrates the performance of the SpinalZFNet, a classifier handling multiple classes. The curves for individual classes: class 0 cyst, class 1 normal, class 2 stone, and class 3 tumor, all show high AUC values, with classes 1 and 3 achieving perfection with an AUC of 1.00%. This indicates an exceptional discriminative power where true positives are maximized, and false positives are minimized or nonexistent, especially for classes 1 and 3. Additionally, the micro-average and macro-average ROC curves indicate a high overall performance across classes, with both curves achieving AUCs around 0.995%, signifying the model’s consistent and robust classification ability. The dashed line represents the baseline of a random chance classifier, which all the individual ROC curves significantly outperform, demonstrating the classifier’s effectiveness.

**Table 2:** Comparative analysis of SpinalZFNet with state-of-the-arts models

Variations	Parameters	VGG + DN with KNN	DenseAUXNet201	MLP-ANN	DRDC	SpinalZFNet
Learning set	Sensitivity(%)	76.4	80.5	80.9	86.7	**98.9**
	Specificity(%)	72.3	72.5	72.5	76.4	**98.8**
	Precision(%)	73.3	74.6	74.6	78.6	**97.7**
	Accuracy(%)	74.5	76.0	75.4	78.4	**98.4**
	F1-Score(%)	74.9	77.5	77.7	82.5	**98.0**
*K*-Fold	Sensitivity(%)	81.7	84.5	85.0	89.4	**99.9**
	Specificity(%)	73.0	79.3	79.4	81.0	**99.6**
	Precision(%)	74.6	80.3	80.4	82.4	**99.6**
	Accuracy(%)	80.5	80.7	82.1	83.5	**99.8**
	F1-Score(%)	78.0	82.4	82.7	85.8	**99.7**

Bold values highlight the highest value

**Fig. 10:**
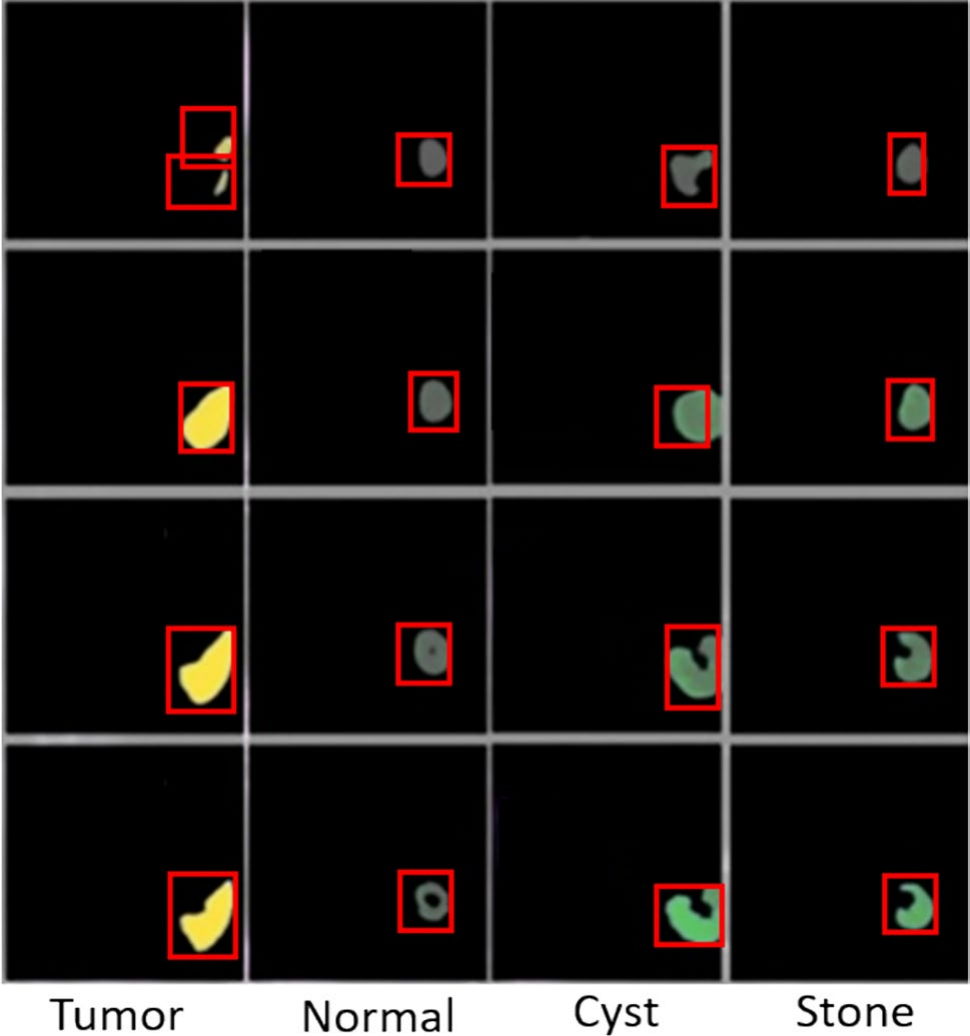
A sample visualizations of the ZFNet model

The sample visualization of the ZFNet model process represents in Fig. 10. The following figure shows the process of training the ZFNet model and categorizing the images based on extracted features, which are highlighted by the model to explain how the procedure helps to classify the disease necessary for classifying the images in training.

The performance comparison of the proposed SpinalZFNet with state-of-the-art models is provided in Table 3. The SpinalZFNet achieves this superior performance with significantly fewer parameters and lower computational costs than other state-of-the-art models. However, SpinalNet demonstrates even better efficiency than the proposed model SpinalZFNet.

**Table 3:** Performance comparative analysis of proposed SpinalZFNet with state-of-the-arts models

Architecture	Parameters (millions)	FLOPs (billions)	Training time (h)	Inference time (ms)
VGG + DN with KNN	138	19.6	12	50
DenseAUXNet201	18	4.0	10	30
MLP-ANN	60	12.0	15	45
DRDC	45	9.0	13	35
SpinalZFNet	23	5.2	9	28
SpinalNet	**5**	**1.5**	**8**	**25**

Bold values highlight the highest value

## Conclusion

This research introduces a hybrid deep learning model, SpinalZFNet, to classify kidney disease accurately from CT images. The median filter is initially used to pre-process the acquired input CT images, and the pre-processed image is segmented using ENet. The segmented image further allows for image augmentation, and the different features are extracted from the augmented image using feature extractors. Finally, the SpinalZFNet is used to classify kidney disease into four distinct categories: normal, cyst, tumor, and stone. Moreover, we evaluated the model’s performance using preferred quality metrics. Our SpinalZFNet outperforms the existing models and achieved 99.9% sensitivity, 99.6% specificity, 99.8% accuracy, and 99.7% F1-Score. Our experimental results prove that the SpinalZFNet can accurately classify four different classifications of kidney disease using CT images.

## Data Availability

The publicly available dataset used in this manuscript.
